# Erasure decoding of convolutional codes using first-order representations

**DOI:** 10.1007/s00498-021-00289-9

**Published:** 2021-06-04

**Authors:** Julia Lieb, Joachim Rosenthal

**Affiliations:** Institute of Mathematics, University of Zurich, Winterthurerstrasse 190, 8057 Zurich, Switzerland

**Keywords:** Convolutional codes, Linear systems, Decoding, Erasure channel

## Abstract

It is well known that there is a correspondence between convolutional codes and discrete-time linear systems over finite fields. In this paper, we employ the linear systems representation of a convolutional code to develop a decoding algorithm for convolutional codes over the erasure channel. In this kind of channel, which is important due to its use for data transmission over the Internet, the receiver knows if a received symbol is correct. We study the decoding problem using the state space description of a convolutional code, and this provides in a natural way additional information. With respect to previously known decoding algorithms, our new algorithm has the advantage that it is able to reduce the decoding delay as well as the computational effort in the erasure recovery process. We describe which properties a convolutional code should have in order to obtain a good decoding performance and illustrate it with an example.

## Introduction

In modern communication, especially over the Internet, the erasure channel is widely used for data transmission. In this type of channel, the receiver knows if an arrived symbol is correct, as each symbol either arrives correctly or is erased. For example, over the Internet messages are transmitted using packets and each packet comes with a check sum. The receiver knows that a packet is correct when the check sum is correct. Otherwise a packet is corrupted or simply is lost during transmission. An especially suitable class of codes for transmission over an erasure channel is the class of convolutional codes [13]. It is known that convolutional codes are closely related to discrete-time linear systems over finite fields, in fact each convolutional code has a so-called input-state-output (ISO) representation via such a linear system [17, 18]. This correspondence was also used in [4–6] to study concatenated convolutional codes. Moreover, the connection between linear systems and convolutional codes was investigated in a more general setup in [22], where multidimensional codes and systems over finite rings were considered.

Hence, decoding of a convolutional code can be viewed as finding the trajectory (consisting of input and output) of the corresponding linear system that is in some sense closest to the received data. The underlying distance measure one uses to identify the closest trajectory (i.e., the closest codeword) depends on the kind of channel that is used for data transmission. This decoding process can also be interpreted as minimizing a cost function attached to the corresponding linear system, which measures the distance of a received word to a codeword or the distance of a measured trajectory to a possible trajectory, respectively. For the Euclidean metric over the field of real numbers ${\mathbb {R}}$, this is nothing else than solving the classical LQ problem, i.e., minimizing the cost function $\sum _{i=0}^{N-1}||u_i-{\hat{u}}_i||^2+||y_i-{\hat{y}}_i||^2$, where ${\hat{u}}\in ({\mathbb {R}}^m)^N$ and ${\hat{y}}\in ({\mathbb {R}}^p)^N$ are received and one wants to find an input $u\in ({\mathbb {R}}^m)^N$ and corresponding output $y\in ({\mathbb {R}}^p)^N$ of the linear system such that this cost function is minimized. This problem is relatively easy to solve, and it is known how to approach it for quite some time, see, e.g., [10, Chapter 3.5.3].

However, for the setting of classical coding theory, where usually the Hamming metric over finite fields is used, it turns out to be in general a hard problem to minimize the corresponding cost function $\sum _{i=0}^{N-1}wt(u_i-{\hat{u}}_i)+wt(y_i-{\hat{y}}_i)$ with ${\hat{u}},u\in ({\mathbb {F}}^m)^N$ and ${\hat{y}},y\in ({\mathbb {F}}^p)^N$ for some finite field ${\mathbb {F}}$. The methods used to solve the LQ problem cannot be applied since the Hamming metric is not induced by a positive definite scalar product. However, the problem becomes much easier for transmission over an erasure channel as done with convolutional codes in this paper. In this setting, one introduces an additional symbol $*$ that stands for an erasure and considers ${\mathbb {F}}\cup \{*\}$ as set of symbols for the decoding. The Hamming metric can easily be extended to this new symbol space, and we are going to minimize the same cost function. The big advantage when decoding over an erasure channel is that we know that all received symbols, i.e., all symbols except $*$ in ${\hat{u}}$ and ${\hat{v}}$, are correct and we only have to find a way to replace the unknowns $*$ be the original values to bring the cost function to its minimal value, which equals the number of erasures. It depends on the number of erasures if unique decoding is possible or if one gets a list of possible codewords. In this paper, we focus on unique decoding, i.e., we present an erasure decoding algorithm that skips part of the sequence if there are too many erasures such that unique decoding is not possible. Our algorithm exploits the ISO representation of a convolutional code via linear systems to recover the erasures in the received sequence. With respect to other erasure decoding algorithms for convolutional codes that can be found in the literature [1, 16, 20], our systems theoretic approach has the advantage that the computational effort and the decoding delay can be reduced.

The paper is structured as follows. In Sect. 2, we give the necessary background on convolutional codes. In Sect. 3, we explain the correspondence of time-discrete linear systems and convolutional codes. In Sect. 4, we present our decoding algorithm, describe which properties a convolutional code should have to perform well with our algorithm and illustrate it with an example. In Sect. 5, we describe the advantages of our algorithm, and in Sect. 6, we conclude with some remarks.

## Convolutional codes

In this section, we start with some basics on convolutional codes.

### Definition 1

An (*n*, *k*) convolutional code $\mathcal C$ is defined as an $\mathbb F[z]$-submodule of ${\mathbb {F}}[z]^n$ of rank *k*. As ${\mathbb {F}}[z]$ is a principal ideal domain, every submodule is free and hence, there exists a full column rank polynomial matrix $G(z) \in \mathbb F[z]^{n \times k}$ whose columns constitute a basis of $\mathcal C$, i.e.,

$$\begin{aligned} \mathcal{C}= & {} Im_{{\mathbb {F}}[z]}G(z) \\= & {} \{G(z)u(z) \, | \, u(z) \in {\mathbb {F}}[z]^k\}. \end{aligned}$$

Such a polynomial matrix *G* is called a **generator matrix** of $\mathcal C$. A basis of an ${\mathbb {F}}[z]$-submodule of $\mathbb F[z]^n$, and therefore also a generator matrix of a convolutional code, is not unique. If *G*(*z*) and ${\tilde{G}}(z)$ in ${\mathbb {F}}[z]^{n \times k}$ are two generator matrices of $\mathcal C$, then one has $G(z)=\tilde{G}(z)U(z)$ for some unimodular matrix $U(z)\in \mathbb F[z]^{k\times k}$ (a unimodular matrix is a polynomial matrix with a polynomial inverse).

Another important parameter of a convolutional code is its **degree** $\delta$, which is defined as the highest (polynomial) degree of the $k\times k$ minors of any generator matrix *G*(*z*) of the code. An (*n*, *k*) convolutional code with degree $\delta$ is denoted as $(n,k,\delta )$ convolutional code. If $\delta _1,...,\delta _k$ are the column degrees (i.e., the largest degrees of any entry of a fixed column) of *G*(*z*), then one has that $\delta \le \delta _1+...+\delta _k$. Moreover, there always exists a generator matrix of $\mathcal C$ such that $\delta = \delta _1+...+\delta _k$ and we call such a generator matrix $\mathbf{column} reduced$.

Furthermore, for the use over an erasure channel, it is a crucial property of a convolutional code to be **non-catastrophic**. A convolutional code is said to be non-catastrophic if one (and therefore each) of its generator matrices is right prime, i.e., if it admits a polynomial left inverse. The following theorem shows why this property is so important.

### Theorem 1

[12, Theorem 1.2.8] Let $\mathcal C$ be an (*n*, *k*) convolutional code. Then, $\mathcal C$ is non-catastrophic if and only if there exists a so-called **parity-check matrix** for $\mathcal C$, i.e., a full row rank polynomial matrix $H(z) \in {\mathbb {F}}[z]^{(n-k) \times n}$ such that

$$\begin{aligned} \mathcal{C}= & {} Ker_{{\mathbb {F}}[z]}H(z) \\= & {} \{v(z) \in {\mathbb {F}}[z]^n \, | \, H(z)v(z)=0\}. \end{aligned}$$

Parity-check matrices are common to be used for decoding of convolutional codes over the erasure channel. Recall that, when transmitting over this kind of channel, each symbol is either received correctly or is not received at all. The first decoding algorithm of convolutional codes over the erasure channel using parity-check matrices can be found in [20], variations of it in [1] or [11]. To investigate the capability of error correction of convolutional codes, it is necessary to define distance measures for these codes.

Therefore, we denote by the **Hamming weight** *wt*(*v*) of $v\in {\mathbb {F}}^n$ the number of its nonzero components. For $v(z)\in {\mathbb {F}}[z]^n$ with $\deg (v(z))=r$, we write $v(z)=v_r+\cdots +v_{0}z^{r}$ with $v_t\in {\mathbb {F}}^n$ for $t=0,\ldots ,r$ and set $v_t=0\in {\mathbb {F}}^n$ for $t\not \in \{0,\ldots ,r\}$. For $j\in {\mathbb {N}}_0$, we define the **j-th column distance** of a convolutional code ${\mathcal {C}}$ as

$$\begin{aligned} d_j^c({\mathcal {C}}):=\min _{v(z)\in {\mathcal {C}}}\left\{ \sum _{t=0}^j wt(v_{r-t})\ |\ v_r\ne 0\right\} . \end{aligned}$$

The erasure correcting capability of a convolutional code increases with its column distances, which are upper bounded as the following theorem shows.

### Theorem 2

[8, Proposition 2.2] Let ${\mathcal {C}}$ be an $(n,k,\delta )$ convolutional code. Then, it holds:

$$\begin{aligned} d_j^c ({\mathcal {C}}) \le (n-k)(j + 1) + 1\ \ \text {for}\ \ j\in {\mathbb {N}}_0. \end{aligned}$$

It is well known that the column distances of a convolutional code could reach this upper bound only up to $j=L:=\left\lfloor \frac{\delta }{k}\right\rfloor +\left\lfloor \frac{\delta }{n-k}\right\rfloor$.

### Definition 2

[9, Definition 1.7] An $(n,k,\delta )$ convolutional code ${\mathcal {C}}$ is said to be **maximum distance profile (MDP)** if

$$\begin{aligned} d_j^c({\mathcal {C}})=(n-k)(j+1)+1\quad \text {for}\ j=0,\ldots ,L:=\left\lfloor \frac{\delta }{k}\right\rfloor +\left\lfloor \frac{\delta }{n-k}\right\rfloor \end{aligned}$$

If one has equality for some $j_0\in {\mathbb {N}}$ in Theorem  2, then one also has equality for $j\le j_0$, see [8]. Hence, it is sufficient to have equality for $j=L$ to obtain an MDP convolutional code. The following theorem presents criteria to check if a convolutional code is MDP.

### Theorem 3

[8, Theorem 2.4] Let ${\mathcal {C}}$ have a column reduced generator matrix $G(z)=\sum _{i=0}^{\mu }G_iz^i\in {\mathbb {F}}[z]^{n\times k}$ and parity-check matrix $H(z)=\sum _{i=0}^{\nu }H_iz^i\in \mathbb F[z]^{(n-k)\times n}$. The following statements are equivalent:

(i) $$d_j^c ({\mathcal {C}})=(n-k)(j + 1) + 1$$
(ii) $$G^c_j:=\left[ \begin{array}{ccc} G_0 &{} &{} 0\\ \vdots &{} \ddots &{} \\ G_j &{} \ldots &{} G_0 \end{array}\right] $$ where $G_i\equiv 0$ for $i>\mu$ has the property that every full size minor that is not trivially zero, i.e., zero for all choices of $G_1,\ldots ,G_j$, is nonzero.
(iii) $$H_j^c:=\left[ \begin{array}{ccc} H_0 &{} &{} 0\\ \vdots &{} \ddots &{} \\ H_j &{} \ldots &{} H_0 \end{array}\right] $$ with $H_i\equiv 0$ for $i>\nu$ has the property that every full size minor that is not trivially zero is nonzero.

The erasure decoding capability of an MDP convolutional code is stated in the following theorem.

### Theorem 4

[20, Corollary 3.2] If for an $(n,k,\delta )$ MDP convolutional code ${\mathcal {C}}$, in any sliding window of length at most $(L+1)n$ at most $(L+1)(n-k)$ erasures occur, then full error correction from left to right is possible.

## The linear systems representation of a convolutional code

In this section, we consider discrete-time linear systems of the form

1 $$\begin{aligned} x(\tau +1)&=Ax(\tau )+Bu(\tau ) \nonumber \\ y(\tau )&=Cx(\tau )+Du(\tau ) \end{aligned}$$

with $A\in {\mathbb {F}}^{s\times s}, B\in {\mathbb {F}}^{s\times k}, C\in {\mathbb {F}}^{(n-k)\times s}, D\in {\mathbb {F}}^{(n-k)\times k}$, input $u\in {\mathbb {F}}^k$, state vector $x\in {\mathbb {F}}^s$, output $y\in {\mathbb {F}}^{n-k}$ and $s, \tau \in {\mathbb {N}}_0$. We identify this system with the matrix-quadruple (*A*, *B*, *C*, *D*). The function $T(z)=C(zI-A)^{-1}B+D$ is called **transfer function** of the linear system.

### Definition 3

A linear system (1) is called

- **reachable** if for each $\xi \in {\mathbb {F}}^s$ there exist $\tau _{*}\in {\mathbb {N}}_0$ and a sequence of inputs $u(0), \ldots , u(\tau _*) \in {\mathbb {F}}^k$ such that the sequence of states $0=x(0), x(1), \ldots ,$ $x(\tau _* +1)$ generated by (1) satisfies $x(\tau _* +1)=\xi$.
- **observable** if $u(\tau )={\tilde{u}}(\tau )$ and $y(\tau )={\tilde{y}}(\tau )$ for all $\tau \in {\mathbb {N}}_0$, i.e., $Cx(\tau )=C{\tilde{x}}(\tau )$ for all $\tau \in {\mathbb {N}}_0$ implies $x(\tau )={\tilde{x}}(\tau )$ for all $\tau \in {\mathbb {N}}_0$. This means that the knowledge of the input and output sequences is sufficient to determine the sequence of states.
- **minimal** if it is reachable and observable.

Recall the following well-known characterization of reachability and observability.

### Theorem 5

(Kalman test) A linear system (1) is reachable if and only if the reachability matrix ${\mathcal {R}}(A,B):=[B,AB,\ldots , A^{s-1}B]\in {\mathbb {F}}^{s\times sk}$ satisfies ${\text {rk}}({\mathcal {R}}(A,B))=s$ and observable if and only if the observability matrix $${\mathcal {O}}(A,C)=\left[ \begin{matrix}C\\ \vdots \\ CA^{s-1} \end{matrix}\right] \in {\mathbb {F}}^{(n-k)s\times s}$$ satisfies ${\text {rk}}({\mathcal {O}}(A,B))=s$.

Next, we will explain how one can obtain a convolutional code from a linear system, see [18]. First, for $(A,B,C,D)\in {\mathbb {F}}^{s\times s}\times {\mathbb {F}}^{s\times k}\times {\mathbb {F}}^{(n-k)\times s}\times {\mathbb {F}}^{(n-k)\times k}$, we set

$$\begin{aligned} P(z):=\left[ \begin{array}{ccc} zI-A &{} 0_{s\times (n-k)} &{} -B \\ -C &{} I_{n-k} &{} -D \end{array}\right] . \end{aligned}$$

The set of $$v(z)=\begin{pmatrix} y(z)\\ u(z)\end{pmatrix}\in {\mathbb {F}}[z]^n$$ with $y(z)\in {\mathbb {F}}[z]^{n-k}$ and $u(z)\in {\mathbb {F}}[z]^{k}$ for which there exists $x(z)\in {\mathbb {F}}[z]^s$ with $P(z)\cdot [x(z)\ y(z)\ u(z)]^{\top }=0$ forms a submodule of ${\mathbb {F}}[z]^n$ of rank *k* and thus, an (*n*, *k*) convolutional code, denoted by ${\mathcal {C}}(A,B,C,D)$.

Moreover, if one writes $x(z)=x_0z^{\gamma }+\cdots +x_{\gamma }$, $y(z)=y_0z^{\gamma }+\cdots +y_{\gamma }$ and $u(z)=u_0z^{\gamma }+\cdots +u_{\gamma }$ with $\gamma =\max (\deg (x),\deg (y),\deg (u))$, it holds

$$\begin{aligned} x_{\tau +1}&=Ax_{\tau }+Bu_{\tau } \\ y_{\tau }&=Cx_{\tau }+Du_{\tau }\\ (x_{\tau }^\top , y_{\tau }^\top , u_{\tau }^\top )&=0\ \text {for}\ \tau >\gamma . \end{aligned}$$

Furthermore, there exist $X\in {\mathbb {F}}[z]^{s\times k}, Y\in {\mathbb {F}}[z]^{(n-k)\times k}, U\in {\mathbb {F}}[z]^{k\times k}$ such that ${\text {ker}}(P(z))={\text {im}}[X(z)^{\top }\ Y(z)^{\top }\ U(z)^{\top }]^{\top }$ and $$G(z)=\left[ \begin{matrix} Y(z)\\ U(z)\end{matrix}\right] $$ is a generator matrix for ${\mathcal {C}}$ with $C(zI-A)^{-1}B+D=Y(z)U(z)^{-1}$, i.e., one is able to obtain a factorization of the transfer function of the linear system via the generator matrix of the corresponding convolutional code, and in the case that this convolutional code is non-catastrophic, one even obtains a coprime factorization of the transfer function.

On the other hand, for each $(n,k,\delta )$ convolutional code ${\mathcal {C}}$, there exists $(A,B,C,D)\in {\mathbb {F}}^{s\times s}\times {\mathbb {F}}^{s\times k}\times {\mathbb {F}}^{(n-k)\times s}\times {\mathbb {F}}^{(n-k)\times k}$ with $s\ge \delta$ such that ${\mathcal {C}}={\mathcal {C}}(A,B,C,D)$. In this case, (*A*, *B*, *C*, *D*) is called linear systems representation or input-state-output (ISO) representation of ${\mathcal {C}}$. Besides, one can always choose $s=\delta$. In this case, (*A*, *B*, *C*, *D*) is called a **minimal representation** of ${\mathcal {C}}$.

### Remark 1

In the coding literature state space descriptions were often done in a graph theoretic manner using so-called trellis representations, see, e.g., [7]. However, especially over large finite fields it is hard to algebraically describe a decoding algorithm and hence, a state space description as above is preferred.

The following theorems show how properties of a linear system are related to properties of the corresponding convolutional code.

### Theorem 6

[18, Theorems 2.9 and 2.10] (*A*, *B*, *C*, *D*) is a minimal representation of ${\mathcal {C}}(A,B,C,D)$ if and only if it is reachable.

### Theorem 7

[18, Lemma 2.11] Assume that (*A*, *B*, *C*, *D*) is reachable. Then, ${\mathcal {C}}(A,B,C,D)$ is non-catastrophic if and only if (*A*, *B*, *C*, *D*) is observable.

## Low-delay erasure decoding algorithm using the linear systems representation

In this chapter, we develop our erasure decoding algorithm based on the ISO representation of the convolutional code. Some first ideas on decoding via this representation can already be found in [21]. We adopt some of the ideas presented there and combine them with new ideas to obtain a complete decoding algorithm.

Assume that we have a message $M=[m_0^{\top }\ \cdots \ m_{\gamma }^{\top }]^{\top }\in {\mathbb {F}}^{k(\gamma +1)}$ with $m_i\in {\mathbb {F}}^k$ which is sent at time step *i*. We write this message as $m(z)=\sum _{i=0}^{\gamma }m_{\gamma -i}z^i$ and encode it via a full rank, left prime, column reduced polynomial generator matrix $G(z)=\sum _{i=0}^{\mu }G_{\mu -i}z^i\in {\mathbb {F}}[z]^{n\times k}$ to obtain $v(z)=G(z)m(z)\in {\mathbb {F}}[z]^n$. We write $$v(z)=\begin{pmatrix} y(z)\\ u(z) \end{pmatrix}$$ with $y(z)=\sum _{i=0}^{\mu +\gamma }y_{\mu +\gamma -i}z^i\in \mathbb F[z]^{n-k}$ and $u(z)=\sum _{i=0}^{\mu +\gamma }u_{\mu +\gamma -i}z^i\in \mathbb F[z]^{k}$. As $m_0$ is sent first, we first receive $$\begin{pmatrix} y_0\\ u_0 \end{pmatrix}=G_0m_0$$, in the next time step $$\begin{pmatrix} y_1\\ u_1 \end{pmatrix}=G_1m_0+G_0m_1$$, and so on.

### Remark 2

ISO representations are not the only way to describe a code in terms of a linear system. There exists also the so-called driving variable representation (see [14] or [15] for details), where the message *m*(*z*) is equal to *u*(*z*) (having the input vectors of the system as coefficient vectors) and the codeword *v*(*z*) equals *y*(*z*) (having the output vectors of the linear system as coefficient vectors). In this case, one gets a rational generator matrix, which equals the transfer function of the linear system. But to make sure that the state and the output of the linear system have finite support, one has to impose restrictions on the input *u*(*z*). Since we do not want to put restrictions on the message *m*(*z*), using ISO representations is more suitable for our aims than using driving variable representations.

Moreover, that *u*(*z*) as part of the codeword has to follow algebraic restrictions will be exploited to achieve one of the main advantages of our decoding algorithm, which is introduced by the following proposition.

### Proposition 1

For $w\in {\mathbb {N}}_0$, define $$E_w:=\left[ \begin{array}{ccc}CA^{\gamma +\mu }B &{}\cdots &{} CB\\ \vdots &{} &{} \vdots \\ CA^{\gamma +\mu +w}B &{}\cdots &{} CA^wB\end{array}\right] $$. One has, $E_w\cdot [u_0^\top ,\ldots ,u_{\gamma +\mu }^\top ]^\top =0$ for all $w\in {\mathbb {N}}_0$.

### Proof

The proposition follows from the fact that $u_i=y_i=0$ for $i>\gamma +\mu$, which implies

$$\begin{aligned} CA^{\gamma +\mu +w}Bu_0+\cdots +CA^wBu_{\gamma +\mu }=0 \end{aligned}$$

for $$w\in {\mathbb {N}}_0$$. $$\square $$

Besides the result of Proposition 1, there are more equations that can be exploited for a decoding algorithm, which we will present in the following.

Let (*A*, *B*, *C*, *D*) be the linear systems representation of the convolutional code generated by *G*(*z*). Then, $(y_0, u_0, \ldots , y_j, u_j)$ represents the beginning of a codeword if and only if

2 $$\begin{aligned}&\left[ -I\ \vline \begin{array}{cccc}D &{} 0 &{} \ldots &{} 0\\ CB &{} \ddots &{} \ddots &{} \vdots \\ \vdots &{} \ddots &{} \ddots &{} 0\\ CA^{j-1}B &{} \ldots &{} CB &{} D \end{array}\right] \begin{pmatrix} y_0\\ \vdots \\ y_j\\ \hline u_0\\ \vdots \\ u_j \end{pmatrix}\nonumber \\&=\left[ \begin{array}{ccccccccc}-I &{} D &{} &{} &{} &{} &{} &{} &{} 0\\ 0 &{} CB &{} -I &{} D &{} &{} \\ 0 &{} CAB &{} 0 &{} CB &{} \ddots \\ \vdots &{} \vdots &{} \vdots &{} \vdots &{} \ddots &{} &{} \ddots &{} \\ 0 &{} CA^{j-1}B &{} 0 &{} CA^{j-2}B &{} \ldots &{} 0 &{} CB &{} -I &{} D \end{array}\right] \begin{pmatrix} y_0\\ u_0\\ \vdots \\ y_j\\ u_j \end{pmatrix}=0 \end{aligned}$$

It follows that if we assume that $v_0, \ldots , v_{i-1}$ are known and $v_i$ contains erasures, then we obtain

3 $$\begin{aligned}&\left[ -I\ \vline \begin{array}{cccc}D &{} 0 &{} \ldots &{} 0\\ CB &{} \ddots &{} \ddots &{} \vdots \\ \vdots &{} \ddots &{} \ddots &{} 0\\ CA^{j-1}B &{} \ldots &{} CB &{} D \end{array}\right] \begin{pmatrix} y_i\\ \vdots \\ y_{i+j}\\ \hline u_i\\ \vdots \\ u_{i+j} \end{pmatrix}=\beta \end{aligned}$$

where $\beta$ is a known vector depending on $v_0, \ldots , v_{i-1}$.

Define ${\mathcal {F}}_0:=D$ and $${\mathcal {F}}_j:=\left[ \begin{array}{cccc}D &{} 0 &{} \ldots &{} 0\\ CB &{} \ddots &{} \ddots &{} \vdots \\ \vdots &{} \ddots &{} \ddots &{} 0\\ CA^{j-1}B &{} \ldots &{} CB &{} D \end{array}\right] $$ for $j\ge 1$.

### Theorem 8

[9, Theorem 2.4] The quadruple (*A*, *B*, *C*, *D*) is the linear systems representation of an MDP convolutional code if and only if each minor of ${\mathcal {F}}_L$ which is not trivially zero is nonzero.

### Remark 3

The existence of MDP convolutional codes for all code parameters was shown in [9]. Moreover, there exist general constructions of MDP convolutional codes in terms of generator or parity-check matrices, see, e.g., [3]. There exist no explicit general constructions for MDP convolutional codes in terms of (*A*, *B*, *C*, *D*), but it is possible to compute (*A*, *B*, *C*, *D*) from the generator matrix of the code (see, e.g., [17]).

Next, we will present equations that will help us to restart the decoding process in case that there are too many erasures for recovery using the preceding equations. For $i,j,l\in {\mathbb {N}}_0$, one has

4 $$\begin{aligned} \left[ \begin{matrix} C\\ \vdots \\ CA^j \end{matrix}\right] x_{i+l}+\left[ -I\ \vline \begin{array}{cccc}D &{} 0 &{} \ldots &{} 0\\ CB &{} \ddots &{} \ddots &{} \vdots \\ \vdots &{} \ddots &{} \ddots &{} 0\\ CA^{j-1}B &{} \ldots &{} CB &{} D \end{array}\right] \begin{pmatrix} y_{i+l}\\ \vdots \\ y_{i+l+j}\\ \hline u_{i+l}\\ \vdots \\ u_{i+l+j} \end{pmatrix}=0 \end{aligned}$$

where

5 $$\begin{aligned} x_{i+l}=A^{i+l-1}Bu_0+\cdots +Bu_{i+l-1} \end{aligned}$$

We assume that the erasure recovering process has to be done within time delay *T*, i.e., it is necessary that $m_i$ can be recovered after one has received (with possible erasures) $v_0, \ldots , v_i, \ldots , v_{i+T}$.

**Table:** 

Main Decoding Algorithm	
**1**: Set $i=-1$.	
**2**: Set $l=0$. If $v_i$ contains erasures, go to 3 and if $v_i$ contains no erasures, set $i=i+1$ and repeat step 2.	
**3**: Set $j=0$.	
**4**: If $v_i$ can be recovered solving the system of linear equations induced by $[-I\ |\ {\mathcal {F}}_j]$ and $v_i,\ldots ,v_{i+j}$ (see (3)), go to 5, otherwise go to 6.	
**5**: Recover the erasures in $v_i$ (and if possible also erasures in $v_{i+1},\ldots , v_{i+j}$), solving the system of linear equations induced by (3). Replace the erased symbols with the correct symbols and go back to 2.	
**6**: If $j=T$, we go to 7. Otherwise, we set $j=j+1$ and go back to 4.	
**7**: Set $l=l+1$.	
**8**: Set $j=0$.	
**9**: If $x_{i+l}$ can be recovered solving the linear system of equations induced by (4) with $x_{i+l}$ and the erased components of $v_{i+l},\ldots , v_{i+l+j}$ as unknowns, we go to 10. Otherwise, we go to 11.	
**10**: Recover $x_{i+l}$ (and as much as possible of $v_{i+l},\ldots , v_{i+l+j}$) with the help of (4).Set $i=i+l$ and go back to 2.	
**11**: If $j=T$, go back to 7. Otherwise set $j=j+1$ and go back to 9.	

**Proof of correctness of the main decoding algorithm**

Steps 1–6:

In steps 1–6, the algorithm recovers erasures forward with minimal possible delay *j* but with at most time delay $j=T$ as long as this is possible. For this recovery, equation (3) is used and the erased components of $y_i,\ldots ,y_{i+j}, u_i,\ldots , u_{i+j}$ are considered as unknown variables of the system of linear equations. To be able to proceed, it is enough to recover $y_i, u_i$, but of course if it is already here possible to recover further components, this should be done.

Steps 7–11:

If the maximal delay $j=T$ is reached in step 6, i.e., no further recovery within the prescribed time delay is possible with steps 1–6, the algorithm tries to recover the earliest possible of the unknown states of the corresponding linear system to be able to restart the decoding process. As $u_0,\ldots ,u_{i-1}$ are known, the sequence of states is known up to $x_{i}$ and the algorithm first tries to recover $x_{i+1}$ with minimum possible time delay *j*. Only if it is not possible to recover $x_{i+l}$ within the prescribed delay, the algorithm tries to recover later states. For the recovery of $x_{i+l}$, equation (4) is used. Since we do not know $x_{i+l}$, its components belong to the unknowns of this system of linear equations together with the erased components of $v_{i+l},\ldots , v_{i+l+j}$.

If $x_{i+l}$ can be recovered, the recovering process following steps 1–6 can be restarted (even if parts of $v_i,\ldots , v_{i+l-1}$ have to be declared as lost for the moment) since the fact that $x_{t+1}$ and $y_t$ for $t\ge i+l$ can be computed with the knowledge of $x_{i+l}$ and $u_t$ for $t\ge i+l$, implies that the recovery of $u_i,\ldots ,u_{i+l-1}$ is not necessary for the recovery of further symbols (see also (5)). Hence, if at any point a state has been successfully recovered, we can start again at the beginning of the algorithm.

Next, we want to exploit the result of Proposition 1 to finish the decoding process before the end of the codeword sequence is reached, in case the received erasure pattern allows for such an earlier termination. Therefore, we add a termination algorithm to our main decoding algorithm. This termination algorithm receives at any time all known symbols of all $u_i$ from the main algorithm.

Furthermore, ${\tilde{E}}_w$ should denote the submatrix of $E_w$ (as defined in Proposition 1) consisting only of the columns corresponding to components of $[u_0^\top ,\ldots ,u_{\gamma +\mu }^\top ]$ that are not known yet.

**Table:** 

**Termination Algorithm**	
**1**: If there exists $w\in \{1,\ldots ,\delta -1\}$ such that ${\tilde{E}}_w$ has full column rank, stop the main decoding algorithm and go to step 2 of the termination algorithm, otherwise wait until more symbols from the main algorithm are obtained.	
**2**: Use the system of linear equations induced by the equation $E_w\cdot [u_0^\top ,\ldots ,u_{\gamma +\mu }^\top ]^\top =0$ to recover all erased components of $[u_0^\top ,\ldots ,u_{\gamma +\mu }^\top ]^\top$. Afterward, use (2) to obtain $[y_0^\top ,\ldots ,y_{\gamma +\mu }^\top ]^\top$.	

**Proof of correctness of the termination algorithm**

Each time, when new symbols arrive or some symbols have been successfully recovered, ${\tilde{E}}_w$ is updated in step 1 and it is checked if there are already enough symbols known to recover the whole message with step 2. Due to the theorem of Cayley–Hamilton, one only has to check ${\tilde{E}}_w$ up to $w=\delta -1$, i.e., the number of iterations is finite.

The equation $E_w\cdot [u_0^\top ,\ldots ,u_{\gamma +\mu }^\top ]^\top =0$ can be written as ${\tilde{E}}_w\cdot {\tilde{u}}=\alpha$, where ${\tilde{u}}$ is the vector consisting of all components of $[u_0^\top ,\ldots ,u_{\gamma +\mu }^\top ]^\top$ that are unknown and $\alpha$ can be computed with the help of the known components of $[u_0^\top ,\ldots ,u_{\gamma +\mu }^\top ]^\top$. If ${\tilde{E}}_w$ has full column rank, the corresponding system of linear equations can be solved and all components of ${\tilde{u}}$ can be recovered.

Note that the termination algorithm works independently of delay constraints and recovers all symbols, i.e., also symbols that had to be declared as lost in steps 7–11 of the main decoding algorithm. However, these symbols are recovered with delay larger than *T*. On the other hand, it is possible that the termination algorithm also recovers symbols that did not even arrive yet, i.e., in some sense it allows to recover some symbols with “negative delay.”

In order to have a good performance for our algorithm, a convolutional code should fulfill the following properties as good as possible:

1. The non-trivial minors of ${\mathcal {F}}_j$ are nonzero for $j=1,\ldots ,T$ (important for steps 1–6 of the main algorithm).
2. The non-trivial minors of $$\left[ \begin{matrix} C &{} \vline \\ \vdots &{} \vline &{} {\mathcal {F}}_j\\ CA^j &{} \vline \end{matrix}\right] $$ are nonzero for $j=1,\ldots ,T$ (important for steps 7–11 of the main algorithm).
3. For as many sets of columns of $E_w$ as possible, there exists $w=1,\ldots ,\delta -1$ such that these columns are linearly independent (important for the termination algorithm).

It is difficult to ensure that all these three properties are perfectly fulfilled. However, since these properties involve similar matrices, it seems to be a good attempt to construct a convolutional code in such a way that some of the properties are fulfilled, and then check how good the other properties are fulfilled. Clearly, if 2. is perfectly fulfilled, then also 1. Furthermore, there already exist constructions for matrices having all non-trivial minors nonzero (in the literature also referred to as superregular matrices), see, e.g., [2, 8, 21]. Hence, to illustrate the performance of our algorithm with an example, we will construct a convolutional code such that 2. is perfectly fulfilled and then investigate how good 3. and 4. are fulfilled.

### Example 1

We will construct a (5, 3, 2) convolutional code for decoding with maximum delay $T=L=1$. Hence, we want to construct $A, C\in {\mathbb {F}}^{2\times 2}$, $B, D\in {\mathbb {F}}^{2\times 3}$ such that $$\left[ \begin{matrix} C &{} D &{} 0 \\ CA &{} CB &{} D\end{matrix}\right] $$ has all non-trivial minors nonzero for a suitable finite field ${\mathbb {F}}$. We use the construction for superregular matrices from [3] as well as the fact that column permutation preserves superregularity to obtain that

$$\begin{aligned} \left[ \begin{matrix} C &{} D &{} 0 \\ CA &{} CB &{} D\end{matrix}\right] =\left[ \begin{matrix} a^8 &{} a^{16} &{} a &{} a^2 &{} a^4 &{} 0 &{} 0 &{} 0 \\ a^{16} &{} a^{32} &{} a^2 &{} a^4 &{} a^8 &{} 0 &{} 0 &{} 0\\ a^{64} &{} a^{128} &{} a^8 &{} a^{16} &{} a^{32} &{} a &{} a^2 &{} a^4\\ a^{128} &{} a^{256} &{} a^{16} &{} a^{32} &{} a^{64} &{} a^2 &{} a^4 &{} a^8\end{matrix}\right] , \end{aligned}$$

where ${\mathbb {F}}={\mathbb {F}}_{p^N}$ with $N>330$ and *a* is a primitive element of ${\mathbb {F}}$, has the property that all non-trivial minors are nonzero. We immediately obtain

$$\begin{aligned} D=\left[ \begin{matrix}a &{} a^2 &{} a^4\\ a^2 &{} a^4 &{} a^8\end{matrix}\right] \quad \text {and}\quad C=\left[ \begin{matrix} a^8 &{} a^{16} \\ a^{16} &{} a^{32} \end{matrix}\right] \end{aligned}$$

and can compute

$$\begin{aligned} B=C^{-1}(CB)=\left[ \begin{matrix}1 &{} 0 &{} -a^{32}(a^8+1)\\ 0 &{} 1 &{} a^{16}(a^{16}+a^8+1)\end{matrix}\right] \end{aligned}$$

and

$$\begin{aligned} A=C^{-1}(CA)=\frac{1}{a^8-1}\left[ \begin{matrix} a^{64}-a^{112} &{} a^{128}-a^{240} \\ a^{104}-a^{48} &{} a^{232}-a^{112} \end{matrix}\right] . \end{aligned}$$

As *B* is full rank, (*A*, *B*, *C*, *D*) is a minimal ISO representation of an (5, 3, 2) convolutional code ${\mathcal {C}}$ and since ${\mathcal {F}}_1$ is superregular, ${\mathcal {C}}$ is an MDP convolutional code. Hence, in particular, it has to fulfill Theorem 3 (ii), which is not possible if $G_1$ has two columns that are identically zero. Hence, a generator matrix *G* of ${\mathcal {C}}$ has at most one column degree that is equal to zero. Consequently, *G* has column degrees 1, 1, 0 since we assumed it to be a column reduced generator matrix and thus, the column degrees of *G* have to sum up to $\delta =2$. Therefore, we obtain $\mu =1$.

Assume $\gamma =3$ and that we receive the following:

**Table:** 

$y_0$	$u_0$	$y_1$	$u_1$	$y_2$	$u_2$	$y_3$	$u_3$	$y_4$	$u_4$
$*$ $*$	$\surd$ $\surd$ $\surd$	$*$ $*$	$*$ $\surd$ $\surd$	$*$ $*$	$\surd$ $\surd$ $\surd$	$\surd$ $\surd$	$\surd$ $\surd$ $\surd$	$*$ $*$	$*$ $*$ $*$

where $*$ symbolizes an erasure and $\surd$ a received symbol.

Since ${\mathcal {C}}$ is MDP, it can recover $n-k$ erasures out of *n* symbols or $2(n-k)$ erasures out of 2*n* symbols (assuming that there are no erasures in front of this window of size *n* or 2*n*, respectively). The steps of our algorithm with ${\mathcal {C}}$ and the above erasure pattern would be the following.

First, the algorithm uses (3) with $j=0$ to recover $y_0$. Afterward, one realizes that it is neither possible to recover $y_1$ and $u_1$ with (3) for $j=0$ nor $y_1, u_1, y_2, u_2$ with (3) for $j=1$. The algorithm applies (4) with $i=l=1$ trying to recover $x_2$ and $y_2$, but this is not possible for $j=0$, i.e., not before $v_3$ has arrived. However, the matrix consisting of the first column of $$\left[ \begin{matrix} CA^3B\\ CA^4B \end{matrix}\right] $$ and all columns of $$\left[ \begin{matrix} CB\\ CAB \end{matrix}\right] $$ has nonzero determinant, and one can use the termination algorithm as soon as $v_3$ has arrived, implying that the recovery of $x_2$ is not necessary anymore. It is possible to recover the lost component of $u_1$ as well as $u_4$ before $u_4$ and $y_4$ were even sent, just with the knowledge of the already known symbols of $u_0, u_1, u_2, u_3$ and with the information that $\gamma =3$, i.e., $u_i=y_i=0$ for $i>4$. Then, with the knowledge of $u_0,\ldots ,u_4$, it is also possible to compute the erased components of $y_1$ and $y_4$. In summary, we are able to recover the whole sequence but part of it only with a larger delay than actually allowed. However, we were able to obtain $u_4, y_4$ already one time interval before these vectors were sent, i.e., in some sense with delay $-1$.

To give also an example where state recovery is helpful, we will consider the same code but assume a different erasure pattern, which should be as follows:

**Table:** 

$y_0$	$u_0$	$y_1$	$u_1$	$y_2$	$u_2$	$y_3$	$u_3$	$y_4$	$u_4$	$y_5$	$u_5$
$*$ $*$	$*$ $*$ $*$	$*$ $*$	$*$ $*$ $*$	$\surd$ $\surd$	$\surd$ $\surd$ $*$	$*$ $\surd$	$\surd$ $\surd$ $\surd$	$*$ $*$	$\surd$ $\surd$ $*$	$*$ $\surd$	$\surd$ $\surd$ $\surd$

Since $v_0$ cannot be recovered with delay $j=0$ or with delay $j=1$, the algorithm tries to recover $x_1$, which, however, is also not possible with $j=0$ or $j=1$ due to too many erasures. Therefore, the algorithm tries to recover $x_2$, which is not possible with $j=0$ but with $j=1$. $v_0$ and $v_1$ have to be declared as lost but it is possible to continue with the decoding and to obtain the erased components of $v_2$ and $v_3$. Afterward, one realizes that $v_4$ cannot be recovered with $j=0$ but with $j=1$ and then, also $v_5$ can be decoded.

Since $u_0$ and $u_1$ are erased completely having 6 components altogether, these vectors can also not be recovered with the termination algorithm as ${\tilde{E}}_w$ has at most 4 rows. However, state recovery allowed us to restart the recovery process after these two many erasures and hence enabled the decoding of the rest of the sequence.

## Performance analysis

In this section, we will explain the two main advantages of our systems theoretic decoding algorithm with respect to other existing erasure decoding algorithm for convolutional codes, namely the reduced decoding delay and the reduced computational effort.

Our algorithm tries to recover the occurring erasures with smallest possible delay by first trying to do the recovery in a window of size *n*, afterward in a window of size 2*n*, and so on. In contrast to this approach, the decoding algorithm in [20] first tries to decode in the largest possible window of size $(L+1)n$ and only decreases this window if it fails to recover all the erasures in the big window. This implies that the decoding delay is always at least *L*. Moreover, it is computationally less complex and less costly to do several decoding steps in small windows than one decoding step in a larger window whose size is the sum of the sizes of the smaller windows since it is easier to solve several small than one large linear system of equations. The idea of improving this aspect of the algorithm from [20] is not new and can be found in several other papers (see [1, 16]). In the following, we will illustrate the two main advantages that our algorithm has with respect to these papers.

### Reduced complexity due to echelon form of systems of equations

The complexity of our and previous erasure decoding algorithms for convolutional codes is determined by the complexity of solving a linear system with $(n-k)(T+1)$ equations and $e\le (n-k)(T+1)$ unknowns. For a general linear system of equations of these dimensions, the complexity is $O(((n-k)(T+1))^{0.8}\cdot e^2)$ according to [19].

By using our linear systems approach, the systems of equations we have to solve for erasure recovery are parts of linear systems that are already in echelon form, see (3). Due to this form, the erasures in the vectors $y_i,\ldots ,y_{i+T}$ can be neglected for the complexity analysis (as you can obtain any erased component of any $y_i$ that has the possibility to be recovered directly from (3)) and one only has to consider the erasures contained in the vectors $u_i,\ldots , u_{i+T}$. If we write $e=e_1+e_2$ where $e_1$ denotes the number of erasures in $y_i,\ldots ,y_{i+T}$ and $e_2$ denotes the number of erasures in $u_i,\ldots ,u_{i+T}$, this reduces the complexity to $O(((n-k)(T+1))^{0.8}\cdot e_2^2)$. Especially, when we transmit over a channel with a statistic that implies that it is more likely to get erasures in the $y_i$ than in the $u_i$, this is an enormous reduction of complexity since we obtained that the decoding complexity is quadratic in the number of erasures.

### Reduced decoding delay due to termination algorithm

As we already observed in our example, the use of the termination algorithm can make it possible to obtain symbols that were not even sent yet, i.e., in some sense we are able to "look into the future" and terminate the decoding before the end of the transmission. This is of course an additional considerable reduction of the decoding delay.

## Conclusion

In this paper, we presented an erasure decoding algorithm for convolutional codes employing their linear systems representation. We observed that this algorithm is able to reduce the decoding delay and the computational effort in comparison with previous algorithms.
